# Surveillance for diseases, pathogens, and toxicants of muskrat (*Ondatra zibethicus*) in Pennsylvania and surrounding regions

**DOI:** 10.1371/journal.pone.0260987

**Published:** 2021-12-09

**Authors:** Laken S. Ganoe, Justin D. Brown, Matthew J. Lovallo, Michael J. Yabsley, Kayla B. Garrett, Alec T. Thompson, Robert H. Poppenga, Mark G. Ruder, W. David Walter

**Affiliations:** 1 Pennsylvania Cooperative Fish and Wildlife Research Unit, The Pennsylvania State University, University Park, Pennsylvania, United States of America; 2 Department of Veterinary and Biomedical Sciences, The Pennsylvania State University, University Park, Pennsylvania, United States of America; 3 Bureau of Wildlife Management, Pennsylvania Game Commission, Harrisburg, Pennsylvania, United States of America; 4 Southeastern Cooperative Wildlife Disease Study, University of Georgia, Athens, Georgia, United States of America; 5 Warnell School of Forestry and Natural Resources, University of Georgia, Athens, Georgia, United States of America; 6 California Animal Health and Food Safety Laboratory, University of California, Davis, California, United States of America; 7 U.S. Geological Survey, Pennsylvania Cooperative Fish and Wildlife Research Unit, The Pennsylvania State University, University Park, Pennsylvania, United States of America; Agricultural Research Service, UNITED STATES

## Abstract

Using diagnostic data and contemporary sampling efforts, we conducted surveillance for a diversity of pathogens, toxicants, and diseases of muskrats (*Ondatra zibethicus*). Between 1977 and 2019, 26 diagnostic cases were examined from Kansas and throughout the Southeast and Mid-Atlantic, USA. We identified multiple causes of mortality in muskrats, but trauma (8/26), Tyzzer’s disease (5/6), and cysticercosis (5/26) were the most common. We also conducted necropsies, during November 2018—January 2019 Pennsylvania muskrat trapping season, on 380 trapper-harvested muskrat carcasses after the pelt was removed. Tissue samples and exudate were tested for presence of or exposure to a suite of pathogens and contaminants. Gastrointestinal tracts were examined for helminths. Intestinal helminths were present in 39.2% of necropsied muskrats, with *Hymenolepis* spp. (62%) and echinostome spp. (44%) being the most common Molecular testing identified a low prevalence of infection with *Clostridium piliforme* in the feces and *Sarcocystis* spp. in the heart. We detected a low seroprevalence to *Toxoplasma gondii* (1/380). No muskrats were positive for *Francisella tularensis* or *Babesia* spp. Cysticercosis was detected in 20% (5/26) of diagnostic cases and 15% (57/380) of our trapper-harvested muskrats. Toxic concentrations of arsenic, cadmium, lead, or mercury were not detected in tested liver samples. Copper, molybdenum, and zinc concentrations were detected at acceptable levels comparative to previous studies. Parasite intensity and abundance were typical of historic reports; however, younger muskrats had higher intensity of infection than older muskrats which is contradictory to what has been previously reported. A diversity of pathogens and contaminants have been reported from muskrats, but the associated disease impacts are poorly understood. Our data are consistent with historic reports and highlight the wide range of parasites, pathogens and contaminants harbored by muskrats in Pennsylvania. The data collected are a critical component in assessing overall muskrat health and serve as a basis for understanding the impacts of disease on recent muskrat population declines.

## Introduction

Wildlife disease surveillance is important for understanding wildlife health and can also provide insight into human and domestic animal health [1]. Monitoring wildlife for diseases can be used to initiate preventative measures or management efforts against outbreaks in other wildlife, domestic animals, and humans. Passive and active methods of surveillance are used for wildlife diseases. Passive surveillance involves investigation of mortality events to determine the cause(s) of disease [1]. Results of passive surveillance give researchers insight into what is currently causing or has previously caused morbidity and mortality. Active surveillance consists of targeted investigations of pathogens, toxicants, and diseases through systematic collections of animals or their samples [2]. Through active surveillance, researchers can assess current and future risk to outbreaks and diseases. While passive surveillance provides identification of causes of mortality, often times screening for a pathogen, toxicant, or disease does not occur if it is not suspected to be the cause. A combination of both passive and active surveillance presents a more complete picture of diseases, pathogens, and contaminants in a species.

Muskrat (*Ondatra zibethicus*) populations are in decline across much of their natural range in North America [3]. The cause(s) of these declines are not fully understood, but some proposed contributing factors include habitat loss and/or degradation, predation, changes in hydrology, and disease [3]. The impacts of disease on muskrat declines are currently unknown and research to address this question is limited by a lack of data on diseases, pathogens, and contaminants in this species. For species such as muskrat, there are unique challenges to passive disease surveillance due to their subterranean dwelling habits, semi-aquatic ecology, and the rapid consumption of their carcasses by predators. Muskrats are a frequently harvested furbearer across the United States and Canada, providing an avenue for active surveillance for pathogens and contaminants [4].

Historically, muskrats have been documented as hosts and reservoirs for a wide range of pathogens and contaminants, however, the relationship between the prevalence of infection or contaminant exposure and muskrat health is poorly understood [5]. Many parasitological studies on muskrats only report prevalence of parasites without addressing body condition or health implications [6–8]. In one study, researchers in southeastern Pennsylvania, USA observed relationships between lead concentrations in muskrat tissue, lead concentrations found in the muskrat food source (i.e., cattails), and muskrat age [9]. Additionally, contaminants from industrial pollution have been loosely linked to disease in other freshwater semi-aquatic mammals (e.g., river otter, *Lontra canadensis*) and have the potential to cause disease in muskrats [10]. Reports on contaminants and impacts of parasites in muskrats are limited which prevents the generation of baseline data to develop acceptable levels or amounts.

In general, the impact of parasites on host health can be exacerbated when co-occurring with other stressors (e.g., contaminants, other pathogens, climate variability) [11]. In response to increasing climate variability, the distribution of aquatic parasites is predicted to shift [12]. This shift is expected to increase contact between parasites and hosts lacking an immunological response to infection, potentially creating concern for muskrat health [12]. There is limited information on muskrat health in terms of bacterial presence/absence, parasite presence/intensity, and baseline contaminant levels.

The overall goal of this research is to define the pathogens, contaminants and diseases of muskrats in Pennsylvania. Such information can serve as the basis for understanding the ecology and impacts of disease on this declining aquatic furbearer species. Specifically, our objectives were to: 1) analyze diagnostic case data on muskrats submitted for necropsy from the Southeast and Mid-Atlantic regions of the United States from 1977 to 2019, 2) conduct active surveillance for specific pathogens and contaminants in trapper-harvested muskrats from Pennsylvania during the 2018–2019 season, and 3) evaluate landscape and host factors for potential influence on pathogen infection or concentrations of contaminants in tissues.

## Materials and methods

### Passive surveillance

We reviewed the diagnostic records for muskrats submitted to the Southeastern Cooperative Wildlife Disease Study, University of Georgia (Athens, Georgia, USA) for post-mortem examination from 1977 to 2019. We extracted the following data from each report and compiled into a database for analysis: history, year of collection, collection location (i.e., state), number of animals collected, diagnostic tests conducted, significant necropsy findings, and diagnoses.

### Active surveillance

#### Carcass collection

**Fig 1:**
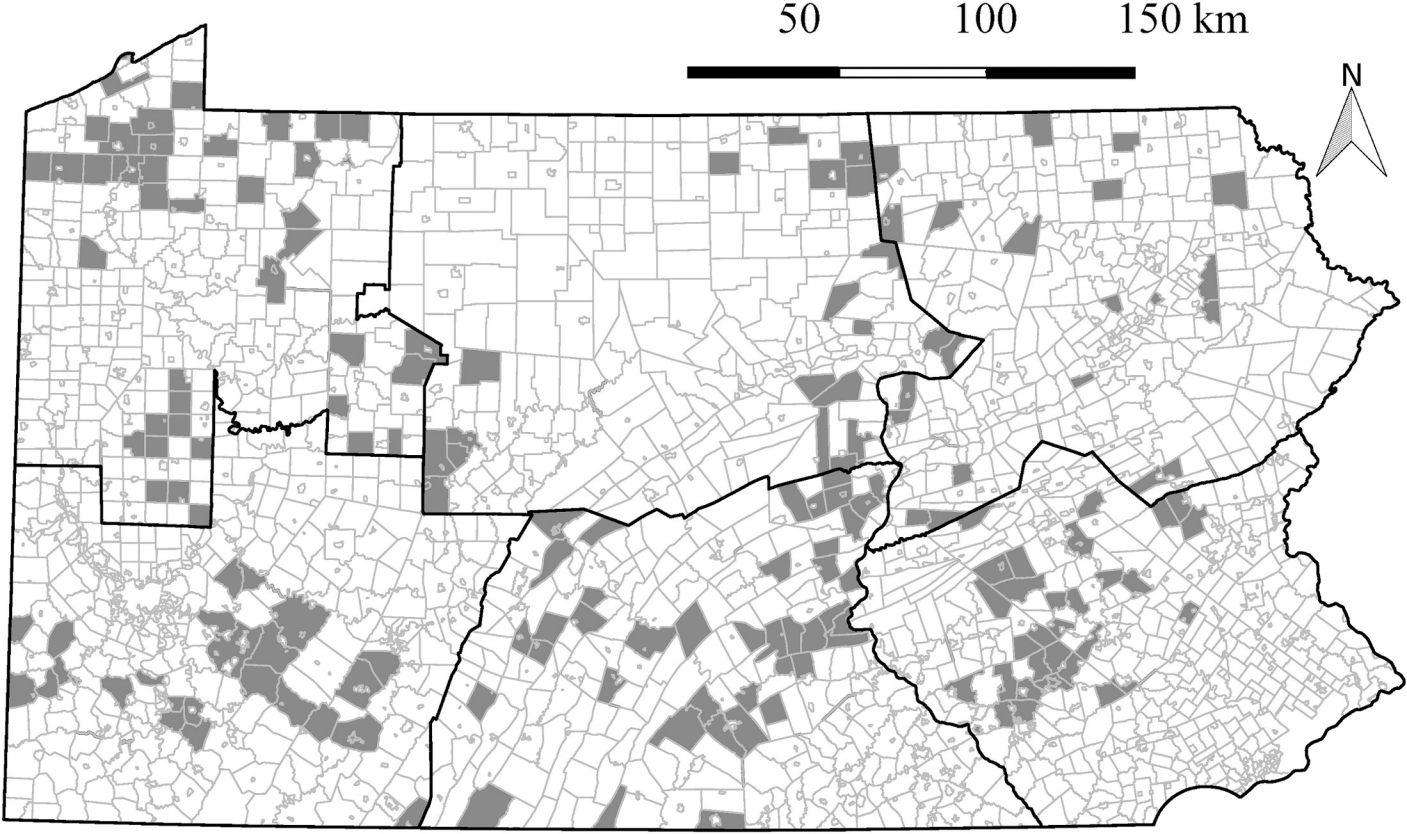
Townships in Pennsylvania, USA where muskrat (*Ondatra zibethicus*) carcasses were collected from trappers in 2018–2019 as indicated by shaded areas. Six regions designated by the Pennsylvania Game Commission are indicated by bold outlines (Game Regions layer provided by Pennsylvania Spatial Data Access [13]).

During the 2018–2019 trapping season, the Pennsylvania Game Commission (PGC) sent out a request to trappers in Pennsylvania for volunteer submission of muskrat carcasses. The trapper removed the pelt and the carcass was frozen until submission to the PGC. Trappers affixed details to each carcass, including name of the trapper, county and township of harvest, harvest date, and details on the location of harvest (e.g., beaver pond near intersection of North Rd and Oak Rd, etc.). From information provided by trappers and satellite imagery, water body type where each muskrat was harvested was classified as one of the following: creek (small tributary), river (>third-order Strahler Order), pond (<1 ha, shallow water body), lake (>1 ha water body), or marsh (water body primarily dominated by reeds and grasses) (hydrology layer provided by Pennsylvania Spatial Data Access). Using satellite imagery, we classified land-cover type (i.e., agricultural, forested, or urban) by the predominant land-cover type (>60% cover) within a 100m buffer around the trapping location (2016 National Land Cover Database). Samples were assigned to their respective PGC region based on the county provided by the trapper (i.e., Northwest [NW], Southwest [SW], Northcentral [NC], Southcentral [SC], Northeast [NE], and Southeast [SE]; Fig 1).

#### Necropsy and tissue collection

Muskrat carcasses were collected from trappers by PGC personnel after the end of the state trapping season. The carcasses were thawed at ambient temperature and necropsied. Prior to dissection, carcass weight was measured, and the sex of the animal was identified. Carcasses submitted with the pelt still intact (n = 7) were not included in any analyses including weight as a variable. The age of each muskrat was not determined as most techniques to determine age are unreliable for this species, apart from pelt primeness, which was not feasible in this study [14]. Carcasses were examined for grossly-visible lesions and parasites. Gastrointestinal content, from stomach to rectum were examined for helminths (e.g., nematodes, cestodes, trematodes) using a 1-mm sieve. Any grossly-visible parasites in visceral tissues (e.g. tapeworm cysts) or intestinal content were counted and preserved in 70% ethanol for further identification via morphologic or molecular methods. Tissues with gross lesions were placed in 10% formalin for fixation. Formalin-fixed tissues were routinely processed for histopathologic examination, they were embedded in paraffin, and cut into 4-μm-thick sections that were stained with hematoxylin and eosin. Uterine tracts were grossly examined at necropsy on all female muskrats to detect and enumerate placental scars. At necropsy, the following tissues were collected from each muskrat for ancillary testing: feces, liver, kidney, heart, tongue, spleen, and skeletal tissue from the thigh. Tissue exudate was also collected from the abdominal cavity of all muskrats, immediately after opening the body cavity, using a syringe and placed into a microcentrifuge tube. All tissues and fluids collected for ancillary testing were placed at –20°C until testing was performed. All sample processing methods were approved by the Institutional Animal Care and Use Committee at The Pennsylvania State University (No. PROTO201800187).

#### Pathogen and toxicant testing

Gastrointestinal helminths (e.g., trematodes, cestodes, nematodes) were identified to the level of family based on morphology. We identified cysticerci in the liver based on morphology of the larvae (size and number of hooks) and analysis of partial cytochrome oxidase subunit I gene sequences (Table 1) [15]. Liver samples were tested for *Babesia* spp. using PCR targeting the 18S rRNA [16]. Tissue exudate samples were screened for antibodies to *Toxoplasma gondii* using a modified agglutination assay [17]. The DNA was extracted from heart samples of muskrats that were seropositive for *T*. *gondii* using a commercial kit (DNeasy Blood and Tissue kit, Qiagen, Hilden, Germany) and tested for apicomplexans using primers targeting a region of the 18S rRNA gene of *T*. *gondii* and related parasites [18]. Due to the frequent detection of *Sarcocystis* spp. using the apicomplexan PCR assay, a second *T*. *gondii*-specific PCR was also conducted to specifically detect *T*. *gondii* (Table 1). Details on the molecular assays used for these pathogens, including primers, targets, and references, is shown in Table 1. Spleen and fecal samples were submitted to the Athens Veterinary Diagnostic Laboratory (Athens, Georgia, USA) for PCR testing for *Francisella tularensis* and *Clostridium piliforme*, respectively.

**Table 1:** Polymerase chain reaction protocols used for detection of selected pathogens in muskrats (*Ondatra zibethicus*).

Pathogen	Gene Target	Primers	Amplicon Size	Reference
Cestode	cytochrome oxidase subunit I	JB3 (5’ TTTTTTGGGCATCCTGAGGTTTAT 3’)	488bp	[15]
		JB4.5 (5’ TAAAGAAAGAACATAATGAAAATG 3’)		
*Babesia*	18S rRNA	5-22F (5’- GTTGATCCTGCCAGTAGT -3’)	1655bp	[16]
		1661R (5’- AACCTTGTTACGACTTCTC -3’)		
*Sarcocystis / Toxoplasma*	18S rRNA	Tg18s58F (5’-CTAAGTATAAGC TTTTATACGGC-3’)	291bp	[19]
		Tg18s 348R (5’-TGCCACG GTAGTCCAATAC-3’)		
*Toxoplasma gondii*	B1	Tox4 (5’- CGCTGCAGGGAGGAAGACGAAAGTTG-3’)	529bp	[18]
		Tox5 (5’- CGCTGCAGACACAGTGCATCTGGATT-3’)		

A subsample of 120 livers (20 livers/PGC region) were submitted to the California Animal Health and Food Safety Laboratory at the University of California Davis (Davis, California, USA) for screening for heavy metals that included: arsenic (As), cadmium (Cd), copper (Cu), iron (Fe), mercury (Hg), manganese (Mn), molybdenum (Mo), lead (Pb), zinc (Zn) and organic chemicals. These 120 livers were selected from subsets of 10 livers from seemingly healthy muskrats (no gross lesions or cysticerci) and 10 livers from muskrats with either, cysticerci, or confirmed cases of infection of *C*. *piliforme*, *F*. *tularensis*, *Babesia spp*., *T*. *gondii*, *or Sarcocystis spp*. within each of the six regions. For analysis of heavy metals, 1 g of liver was digested with 3ml of nitric acid at 190°C. After the digestion was completed, 2 mL of hydrochloric acid was added, and the sample was brought to 10 mL with 18Mohm water. The sample was then analyzed by inductively coupled plasma optical emission spectrometry (ICP-OES). To ensure data quality, a method blank, laboratory control spike, sample over-spike, and a certified reference material (CRM: from the National Research Council of Canada) was digested and analyzed with each batch. For every ten samples, a drift check was also run to ensure instrument stability. The detection limits for each heavy metal were as follows; 1 ppm for Fe, As, Pb, and Hg, 0.4 ppm for Mo, 0.3 ppm for Zn, Cu, and Cd, and 0.1 ppm for Mn. All results were reported based on wet weight of tissue.

Screening for organic chemicals was performed using a combination of gas chromatography-mass spectrometry (GC/MS) and liquid chromatography-mass spectrometry (LC/MS). These screens are designed to detect hundreds of diverse organic compounds from different chemical categories, including pesticides, environmental contaminants, drugs and natural products. For GC/MS screening, liver samples were homogenized in 5% ethanol in ethyl acetate, centrifuged, and a portion of the extract was then evaporated dry and reconstituted in 30% ethyl acetate in hexane. This solution was purified using a gel permeation chromatography system (J2 Scientific, Columbia, MO) equipped with S-X3 Bio-Beads (Bio-Rad, Hercules, CA). Extracts were then analyzed by GC-MS on an Agilent 6890–5975 system. The GC was fitted with a 30 m Agilent DB-5 column. A temperature gradient was used for the analysis with the initial temperature of 40°C held for 5 minutes after injection. It was then ramped at 20°C/minute to 290°C and held at that temperature for 14 minutes. The mass spectrometer was operated in full scan electron ionization mode, scanning from m/z 45 to m/z 650. Automated software was used to detect peaks in the total ion chromatogram and search their spectra against the Wiley mass spectral library (11th Ed., John Wiley and Sons, Hoboken, NJ). Automated Mass Spectral Deconvolution and Identification System deconvolution and library search software (NIST, Gaithersburg, MD) was also used to identify compounds present in the samples. Results generated by these two programs were reviewed by toxicology personnel to determine the presence of toxicants. The LC/MS was conducted as described [20].

### Statistical analysis

Prevalence was defined as the ratio of number of muskrats with respective diagnoses to the total muskrats sampled. For the active surveillance data, we used a one-way analysis of variance (ANOVA) to determine regional and landscape differences in intestinal parasite prevalence. A loess regression was fit to determine the relationship for each sex between parasite abundance (i.e., total number of worms collected in each individual) and carcass weight. A hierarchical Bayesian censored analysis of covariance (ANCOVA) model was used to estimate and compare each heavy metal concentrations among female and male muskrats, while accounting for weight [21]. Additionally, we used this model to compare heavy metal concentrations in the presence and absence of intestinal parasites. The models were as follows:

$$y_{i}∼N(\alpha_{j(i)}∙{Sex}_{i}+\beta_{j(i)}∙{weight}_{i},\sigma_{y}^{2})I(,C_{i}),\;for\;i,\ldots n$$

$$y_{i}∼N(\alpha_{j(i)}∙P_{i}+\beta_{j(i)}∙{weight}_{i},\sigma_{y}^{2})I(,C_{i}),\;for\;i,\ldots n$$

Where *y*_*i*_ is the log_e_-concentration of contaminant observation *i*, *α*_*j*_ is the intercept for each sex (model 1) or parasite prevalence (P; model 2), and *β*_*j*_ is the slope of the log_e_-concentration of contaminant and weight. We used I(, *C*_*i*_) to indicate a censored value for each contaminant with a log_e_-reporting level of *C*_*i*_, where *I*, theoretically, could have its own detection limit for each observation. A diffuse normal prior (N[0, 1000]) was used for the intercepts and slopes and a diffuse uniform prior (U[0,10]) was used for *σ*_*y*_. We ran three parallel Markov chains beginning each chain with random starting values. Each chain was run for 10,000 iterations, from which the first 5,000 were discarded. This resulted in 15,000 samples used to summarize the posterior distribution. Convergence was assessed visually through inspection of trace plots and quantitatively using the Brooks-Gelman-Rubin statistic [22]. Models were fit in the program JAGS [23] using the jagsUI package [24] in program R [25]. Differences in slopes and intercepts between males and females, and between the presence and absence of intestinal parasites were assessed by evaluating if the 95% credible interval of the difference between sex-specific parameters overlapped with zero.

## Results

### Passive surveillance

A total of 26 muskrats from five states in the eastern USA and Kansas were submitted for necropsy to the Southeastern Cooperative Wildlife Disease Study from 1977 to 2019 (Table 2). Trauma was the most common cause of mortality in muskrats (8/26; 31%). Of the eight trauma cases, four (50%) were related to dog attacks, two (25%) were human-associated injuries, one (12.5%) was predation, and one (12.5%) was an unknown source of trauma. Tyzzer’s disease, caused by the systemic bacterium *C*. *piliforme*, was the second most common cause of mortality (5/26; 19%). Three muskrats in Georgia, USA in 1984 had gross lesions on the liver and tested positive for *C*. *piliforme*. Although it was not confirmed, two additional muskrats in Virginia, USA in 1992 were suspected but not confirmed to have the bacteria and mortality was attributed to the disease.

**Table 2:** Passive surveillance and diagnoses for 26 muskrats (*Ondatra zibethicus*) submitted to the Southeastern Cooperative Wildlife Disease Study, 1977–2019.

Diagnosis	n	State in USA	Year (number of cases if >1)
trauma	8	GA, PA, VA	1977, 1981(2), 1987(3), 2008, 2017
Tyzzer’s disease	5	GA, VA	1984, 1992
cysticercosis	5	PA, VA	2006, 2007, 2018
undetermined	3	KS, VA	2006, 2008, 2019
neoplasia	2	GA, MD	1987, 1997
systemic bacterial infection	2	MD, WV	1984, 2012
cellulitis and dermatitis of tail	1	VA	2006

*State abbreviations: Georgia (GA), Pennsylvania (PA), Virginia (VA), West Virginia (WV), Maryland (MD), and Kansas (KS).

Upon review of the diagnostic cases, 12/26 muskrats had parasitic cysts, however, mortalities of only 5 of these individuals were attributed to cystercercosis (Table 2). In the cystercercosis cases, cysts were identified in multiple organs (e.g. liver, lung, kidney, spleen, ovaries, brain, subcutaneous tissue), but were most commonly detected in and most numerous in the liver. Nodules ranged in size (1 mm–4 cm diameter) and quantity (1 to dozens). Although the causative cestode was not definitively identified, four of the cysticercosis cases were caused by *Hydatigera* or *Taenia* species, which is historically the most common reported cause of cysticercosis in muskrats [5]. The fifth case of cysticercosis was in a muskrat from Pennsylvania in 2018. This muskrat had a severe infection with hundreds of much smaller cysts (1–7mm diameter) in multiple organs determined to be *Versteria* sp. based on sequence analysis of the COI gene [26]. Additionally, one muskrat from Maryland had a 3 x 4.5 cm osteogenic tumor attached to the wing of the ilium, however, the postmortem decomposition of the carcass prohibited further investigation of the specimen.

Of the 26 muskrats submitted for diagnostic tests, few were screened for bacterial, viral, or toxic agents. Fourteen cases were not tested for bacterial infection. Those that were tested consisted of screens for *F*. *tularensis* (4/12; 33%), *C*. *piliforme* (4/12; 33%), and general bacterial infection (4/12; 33%). One case returned positive in the general bacterial screen, and identified *Salmonella* sp. Additionally, 4/26 (15%) cases directly related to interaction with domestic animals were screened for viral infection to identify possible viral (e.g., rabies, canine distemper) transmission. In a diagnostic case in Maryland, USA, one carcass with a systemic bacterial infection was also examined for the presence of microcystins following reports of wildlife mortalities, including muskrat, along the Chesapeake Bay shoreline. The muskrat was found over a month into the mortality event that coincided with microcystin blooms. Microcystins were detected in the liver (237 ng/g) and stomach contents (791 ng/g); levels were higher than those previously associated with mammalian mortality events in domestic dog (*Canis familiaris*; 10–20 ng/g), sea otter (*Enhydra lutris*; 11.8–348 ng/g), and cattle (*Bos* sp.; 15 ng/g) (data provided by Peter McGowan of the United States Fish and Wildlife Service).

### Active surveillance

**Fig 2:**
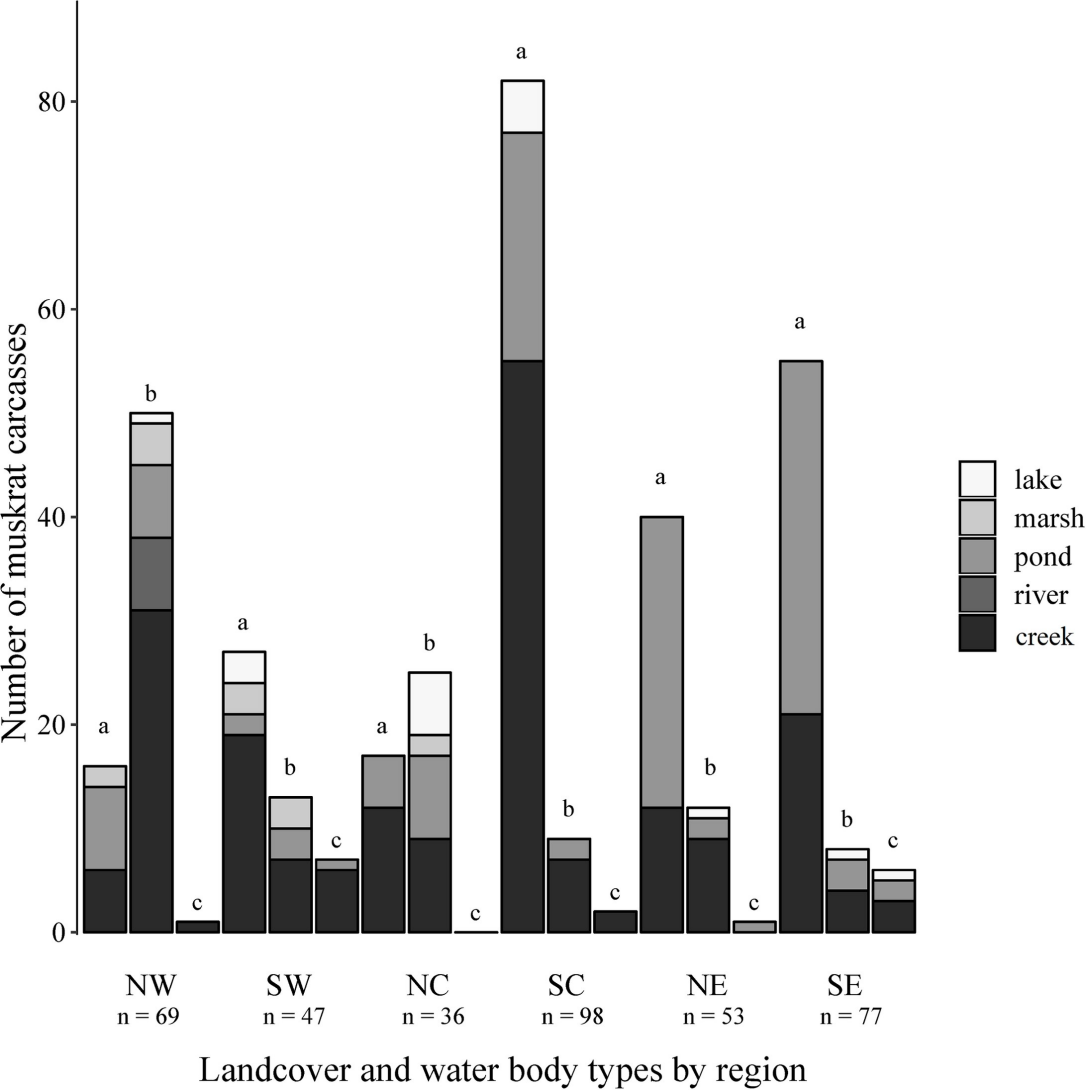
Distribution of landcover type and water body type where muskrats (*Ondatra zibethicus*) were collected in Pennsylvania, USA, 2018–2019. Landcover types were designated by bars within regions and included: agriculture (a), forest (b), and urban (c). Regions within Pennsylvania are Northwest (NW), Southwest (SW), Northcentral (NC), Southcentral (SC), Northeast (NE), and Southeast (SE).

**Fig 3:**
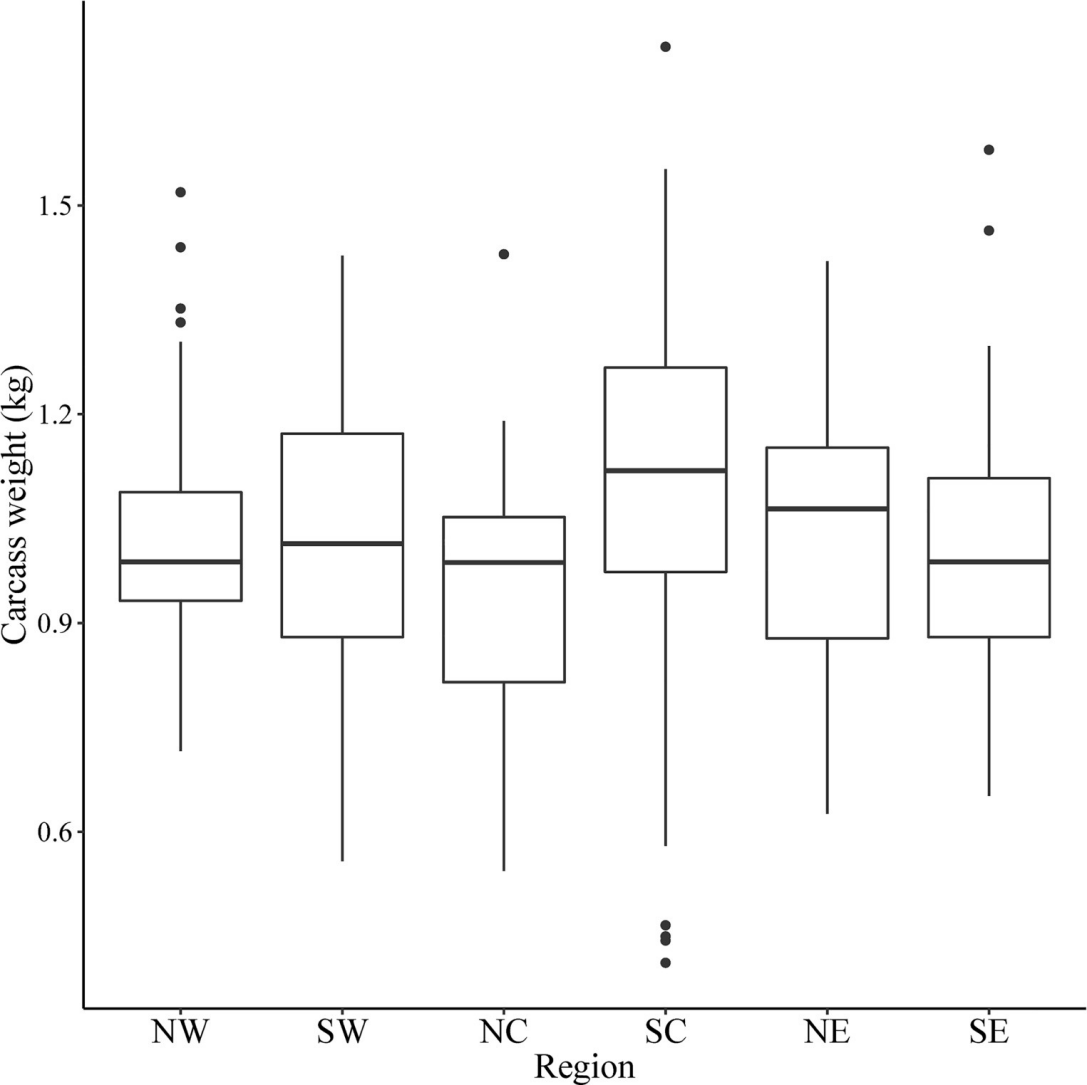
Muskrat (*Ondatra zibethicus*) carcass weight (kg) in each region in Pennsylvania, USA, 2018–2019 (NW-Northwest, SW-Southwest, NC-Northcentral, SC-Southcentral, NE-Northeast, SE-Southeast).

**Fig 4:**
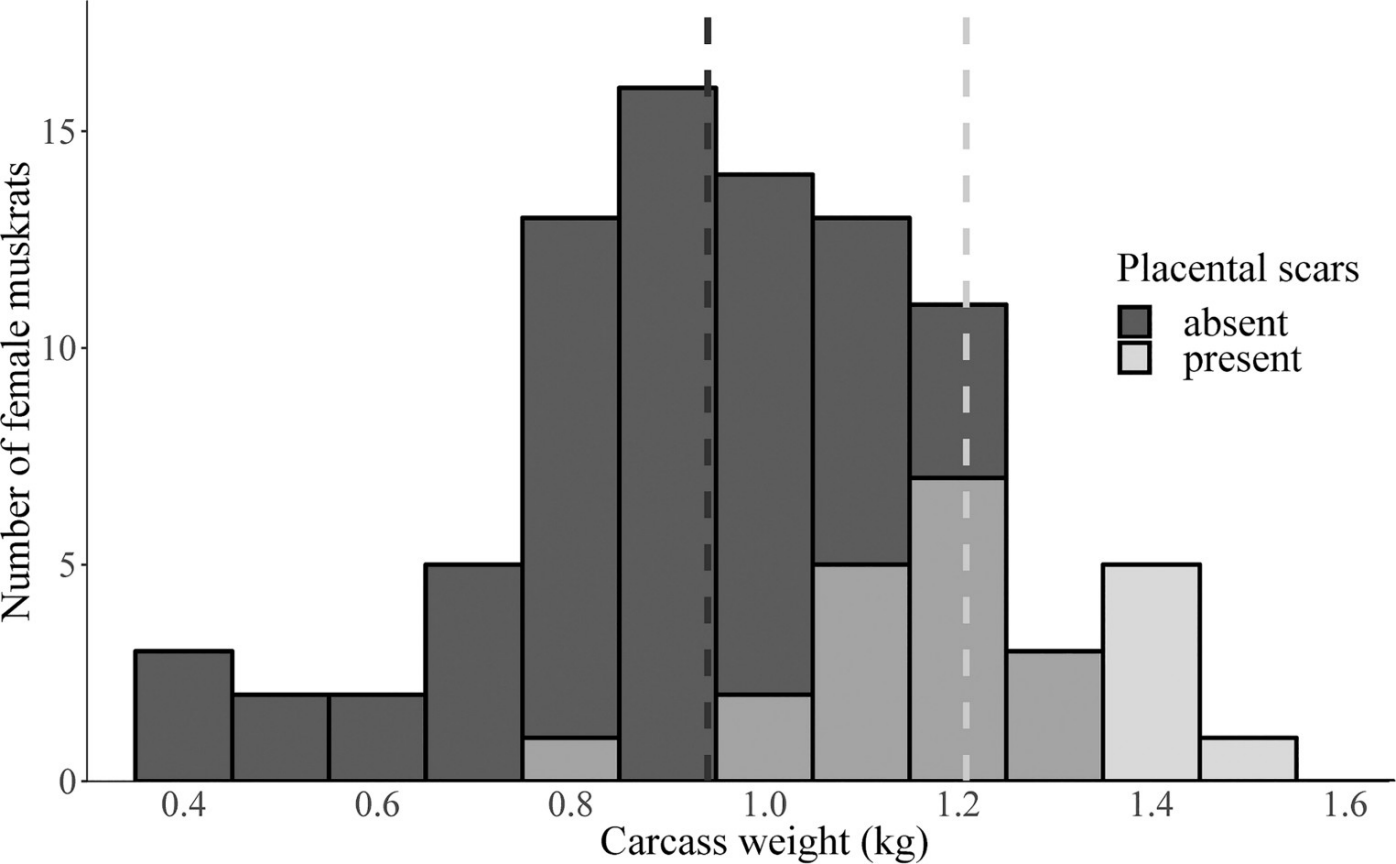
Distribution of female muskrat (*Ondatra zibethicus*) carcass weight (kg) with respect to the presence or absence of placental scars in Pennsylvania, USA, 2018–2019. Mean weight for each group is indicated by dashed lines.

We necropsied 380 muskrat carcasses from across Pennsylvania (Fig 1), comprised of 214 (56.3%) males and 166 (43.7%) females. Muskrats were collected from a diversity of water body types, which varied by region (Fig 2). Creeks (n = 201) and ponds (n = 128) were the most common water body type followed by lakes (n = 18), marshes (n = 14), and rivers (n = 7). Muskrats were collected from three land-cover types: agricultural (n = 227), forested (n = 121), and urban (n = 17). We were unable to determine specific water body type or land-cover type for 15/380 muskrats due to lack of information provided by the trapper. The average carcass weight was 1026.85 g (*SE* = 10.33) with the most variation in weight in the SC region (Fig 3). Males had higher mean carcass weights (1053.83 g, *SE* = 12.99) than females (992.08 g, *SE* = 16.33; *P* = 0.003). Placental scars were identified in 29% (24/166) of females. Females with placental scars had higher mean carcass weights (1208.62 g, *SE* = 34.24) than females without (941.44 g, *SE* = 22.80, *P* < 0.001). Of the 24 females with placental scars, 22 (91.7%) had carcass weights over 1000 g (Fig 4).

**Fig 5:**
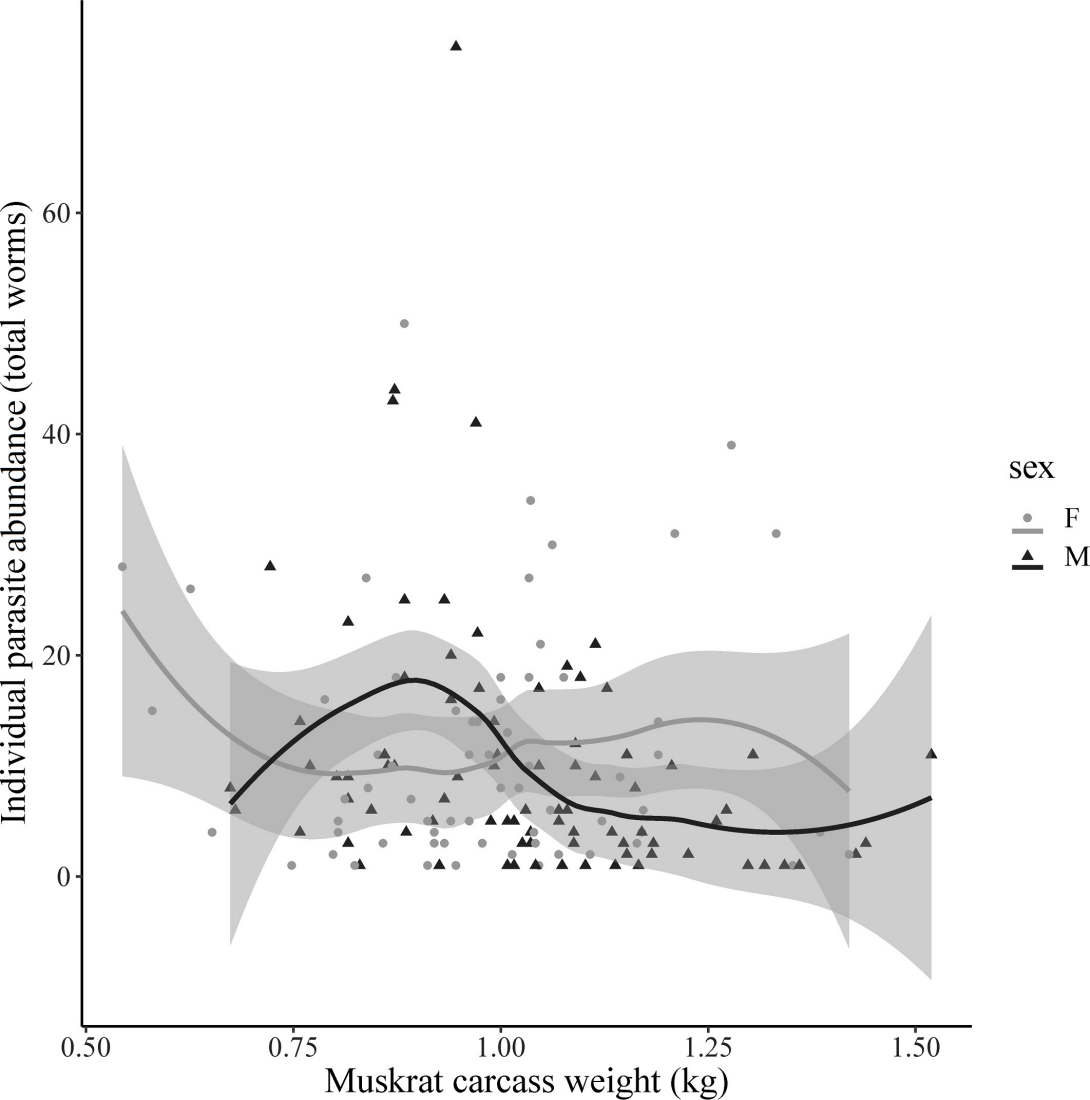
Loess regression of carcass weight (kg) to parasite abundance found in individual muskrats (*Ondatra zibethicus*) for males (M) and females (F) in Pennsylvania, USA in 2019.

**Fig 6:**
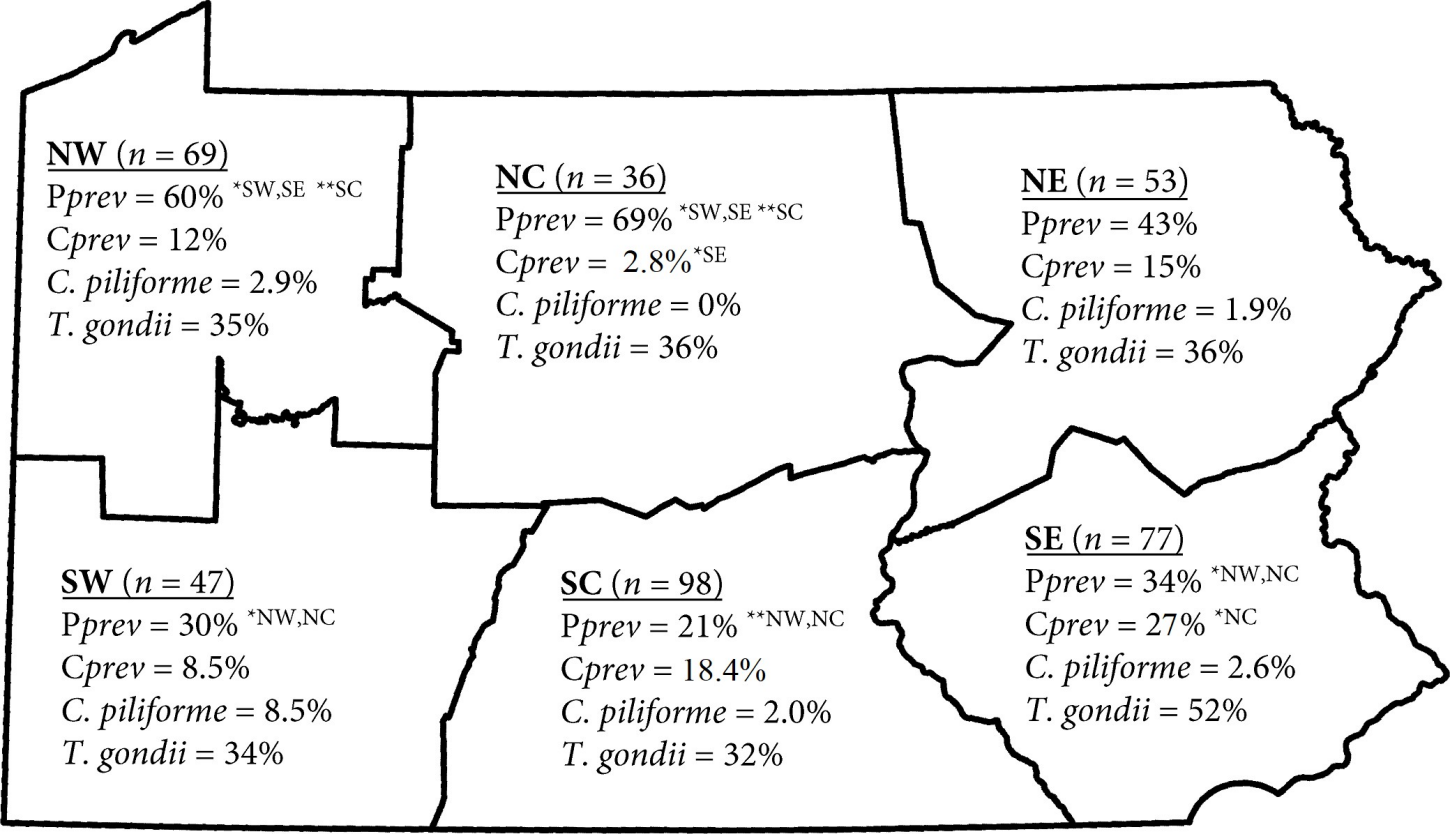
Prevalence of intestinal helminths (P*prev*), cysts (C*prev*), *Clostridium piliforme*, and *Toxoplasma gondii* in muskrats (*Ondatra zibethicus*) by region (NW-Northwest, SW-Southwest, NC-Northcentral, SC-Southcentral, NE-Northeast, SE-Southeast) in Pennsylvania, USA, 2018–2019. * = *P* < 0.001, ** = 0.001 < *P* < 0.05.

We detected intestinal helminths in 149/380 (39.2%) muskrats, of which 83 were males (39% of all males) and 66 were females (40% of all females). The most common intestinal helminths detected were *Hymenolepis* spp. (cestodes) followed by echinostomes (trematodes) in 92/149 (62%) and 66/149 (44%) muskrats, respectively. Co-infections with these parasites were identified in 9 muskrats (S1 Appendix). We detected 632 individual cestodes and 959 individual trematodes, with average intensity of 9.58 worms and 13.89 worms, respectively. No nematode species were detected which may be a result of methodology used to examine intestinal contents. Overall intestinal parasite intensity ranged from 1 to 75 worms ($\bar{x}=10.07$ worms), and intensity was highest in muskrats under 1000 g (Fig 5). Geographically, the NC and NW had a higher prevalence of intestinal parasites than any of the southern regions, SC (*P* < 0.001, *P* < 0.001, respectively), SE (*P* = 0.002, *P* = 0.023, respectively), and SW (*P* = 0.002, *P* = 0.018, respectively) (Fig 6). Forested areas had higher prevalence of intestinal parasites (n = 69/121, *P* < 0.001) than agricultural areas (n = 72/227). Intestinal parasites were found in all water body types with muskrats from lakes having a higher prevalence than muskrats compared with those from ponds (*P* = 0.026) and creeks (*P* < 0.001). Muskrats in ponds had a higher prevalence of intestinal parasites than those in creeks (*P* = 0.017).

Parasitic cysts were observed in livers of 57 (15%) muskrats, with the highest prevalence of cysts occurring in the SE region (21/77; 27%; Fig 6) and the lowest prevalence occurring in the NC region (1/36; 3%). Of the 57 livers with cysts, 55 (97%) had low numbers of cysts (average = 1.98 cysts; range = 1–16), which were large in size (5–22 mm). Based on morphology, the strobilocerci from all but one muskrat were morphologically consistent with *Hydatigera* or *Taenia* spp. The sizes of the large hooks (320–410 μm) and small hooks (190–250 μm) from these strobilicerci were consistent with *H*. *taeniaeformis* (formally called *T*. *taeniaeformis*; S1 Appendix [27]. Sequences of individual strobilicerci from 18 muskrats confirmed that all these large cysts were *H*. *taeniaeformis* (one group of cysts from 6 muskrats was 100% similar to *H*. *taeniaeformis* (Accession number JQ837814), one muskrat had a single nucleotide substitution (131A→G), and the remaining 11 muskrats had a polymorphic base (R) at site 131). The cysticerci from the one remaining muskrat with large cysts and two additional muskrats with dozens of small cysts (1-5mm) were identified as *Versteria* sp. through a combination of morphology and PCR/sequencing [26]. The sequences of these *Versteria* were identical to the one case detected in the passive surveillance and were similar to those from a human from Pennsylvania, mink (*Neovison vison*) and ermine (*Mustela erminea*) from Oregon, USA and Colorado, USA, as well as a captive organutan (*Pongo pygmaeus*) from Wisconsin, USA [28, 29]. All three muskrats with *Versteria* infections were trapped in counties in the NE and SE regions. Incidentally, while microscopically examining cysts from the livers of two muskrats, strings of *Calodium hepaticum* eggs were observed.

Based on PCR testing, none of the 380 muskrat liver samples were positive for *F*. *tularensis* or *Babesia* spp., but 11 of 380 (2.9%) fecal samples were positive for *C*. *piliforme* (Table 3; S1 Appendix). None of the muskrats positive for *C*. *piliforme* had gross or microscopic lesions in the liver consistent with Tyzzer’s disease. Using the modified agglutination assay, 143/380 (38%) of tissue exudate samples were positive for antibodies to *T*. *gondii* (Ab titer>1:25) (Fig 6). Of these 143 muskrats, heart samples from 52 (36%) were positive for apicomplexans by PCR. However, sequencing of a random subset of these amplicons (n = 28), indicated that only one was positive for *T*. *gondii*. One of the remaining muskrats was positive for a *Hammondia* sp. and the sequence was 97.3% identical to *H*. *hammondi* (Accession number AH008381), which is the only *Hammondia* species with 18S rRNA sequences available in GenBank. The remaining 26 (93%) muskrats were positive for a *Sarcocystis* sp. All but one of the *Sarcocystis* sp. sequences were identical and 99.3% similar to *S*. *ratti* reported from black rats (*Rattus rattus)* from Latvia (Accession number MK425190). The final sequence was 98.7% similar to a *Sarcocystis* sp. from a barred owl (*Strix varia*; Accession number MF162315) from the USA.

**Table 3:** Summary of active pathogen surveillance results in muskrats (*Ondatra zibethicus*) collected from Pennsylvania, USA, 2018–2019.

Pathogen	Sample	Assay	No. positive/No tested (% positive)
*Clostridium piliforme*	Feces	PCR	2.89%
*Francisella tularensis*	Liver	PCR	0.00%
*Babesia* spp.	Liver	PCR	0.00%
*Toxoplasma gondii*	Tissue exudate	PCR/Sequencing*	37.70%
	Heart	Serology: Modified Agglutination Assay (MAT)	0.26%
*Sarcocystis* spp.	Heart, Tissue exudate	PRC/Sequencing*	6.84%

*Only muskrats positive for antibodies to *T*. *gondii* were PCR tested using an apicomplexan screening PCR that will amplify various genera of interest including *Toxoplasma*, *Sarcocystis*, and *Hammondia*. For any sample that was sequenced confirmed to have *Sarcocystis* or *Hammondia* we also ran *T*. *gondii*-specific PCR to rule out coinfection.

**Fig 7:**
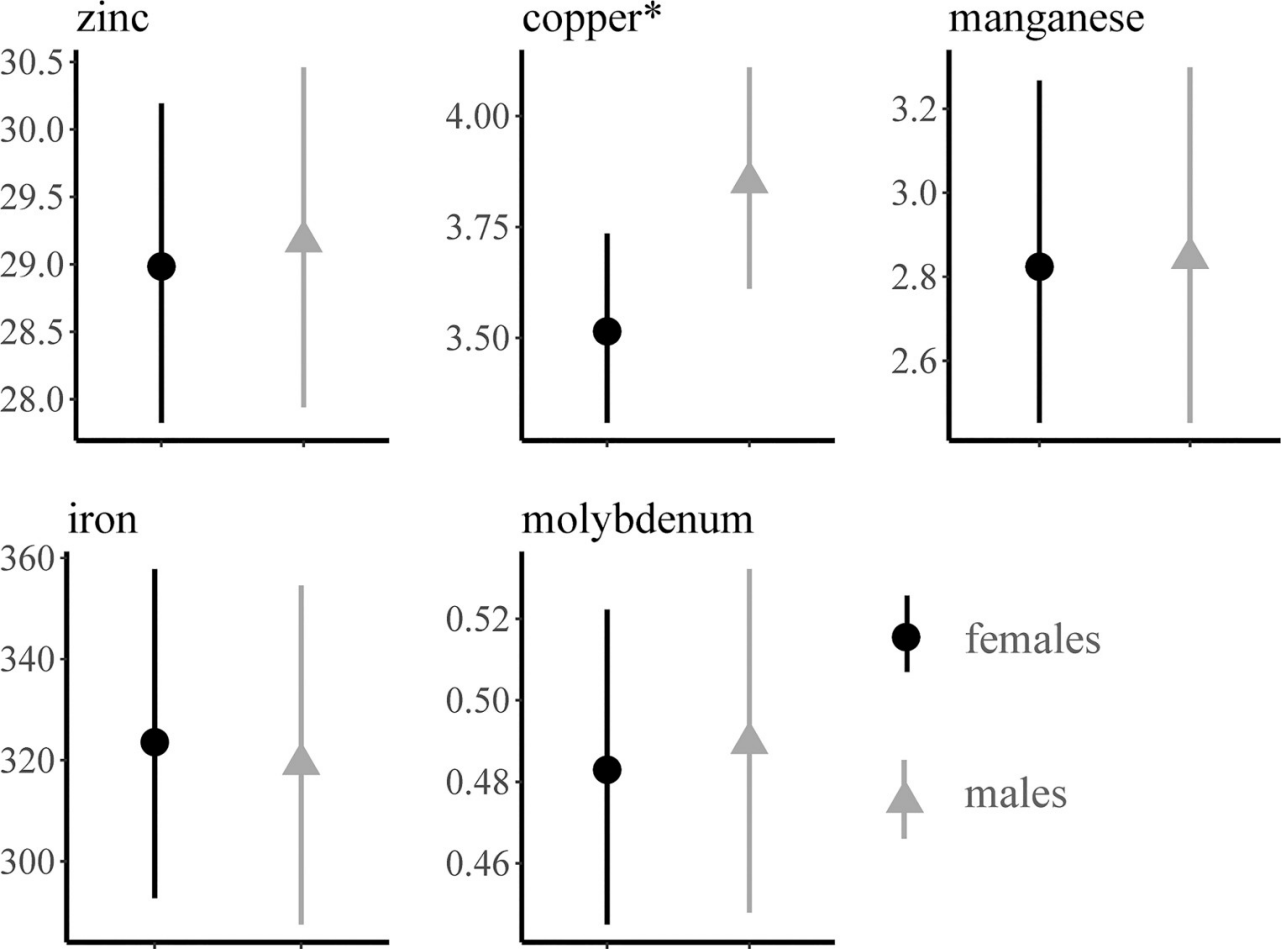
Mean liver concentrations (ppm, wet weight) of heavy metals in male and female muskrats (*Ondatra zibethicus*) in Pennsylvania, USA, 2018–2019. *Confidence interval of the mean difference does not overlap zero.

**Fig 8:**
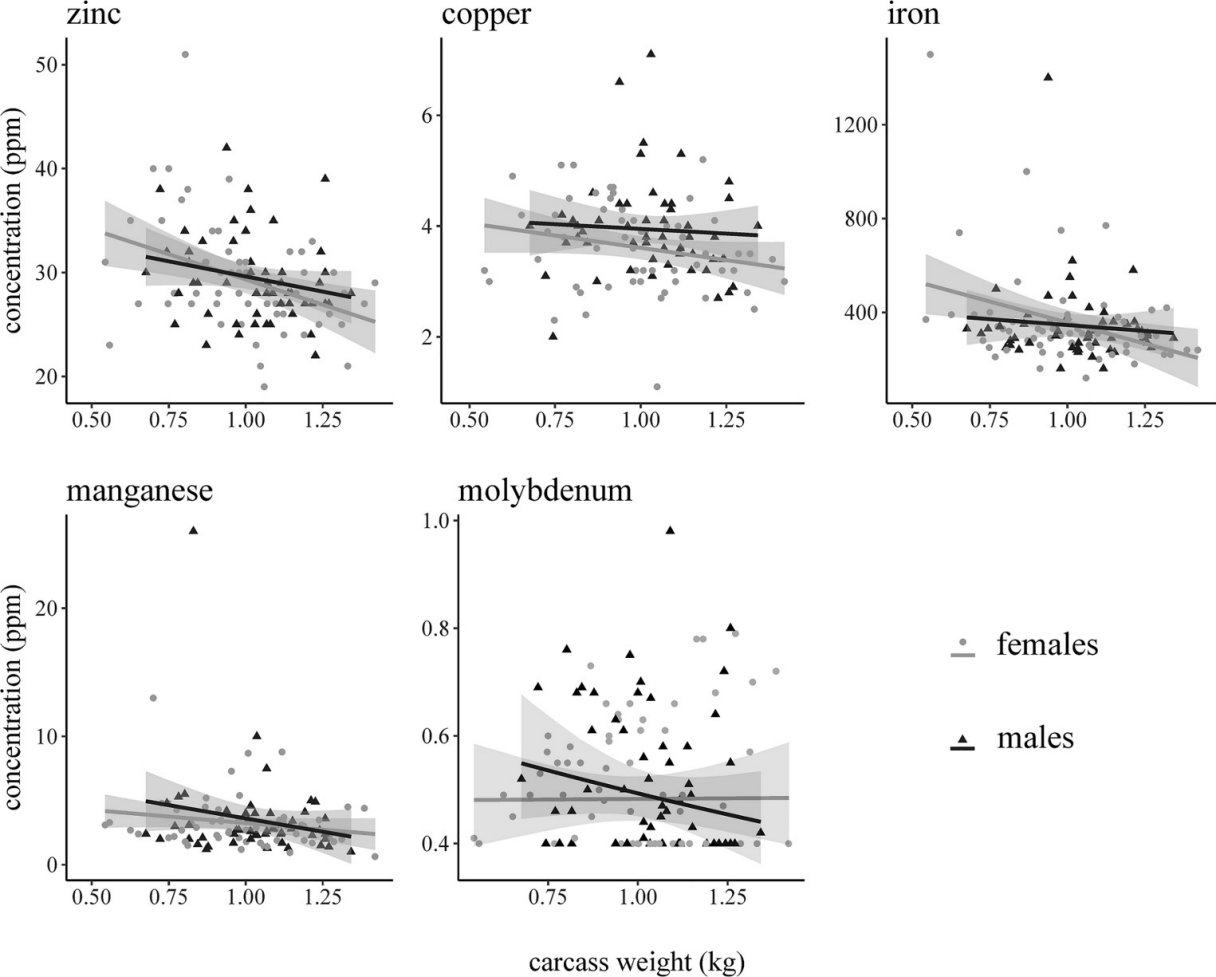
Graphical representation of a Bayesian censored analysis of covariance model depicting relationships between muskrat (*Ondatra zibethicus*) carcass weight (kg) and heavy metal concentrations (ppm, wet weight) for each sex.

**Fig 9:**
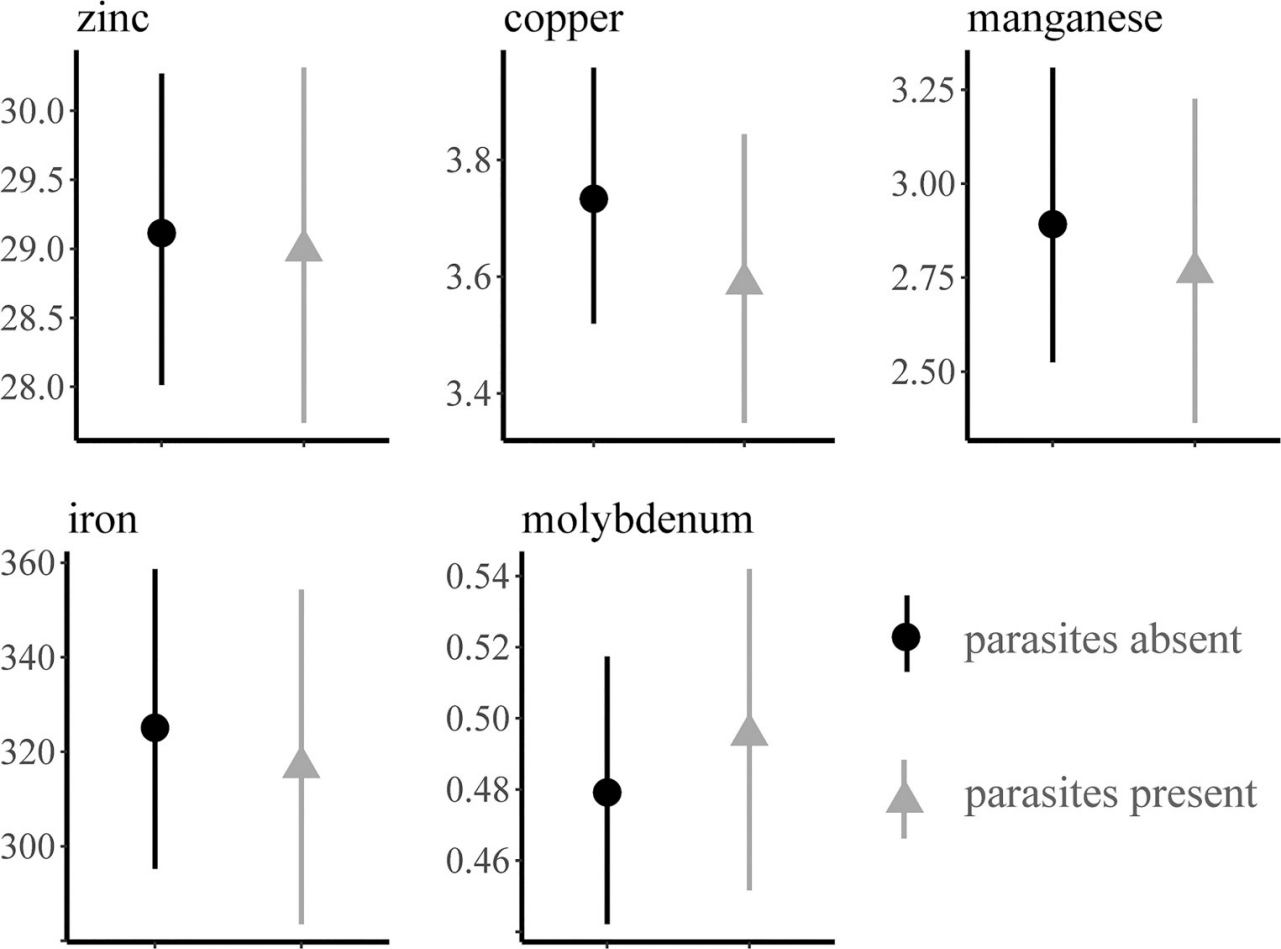
Mean liver concentrations (ppm, wet weight) of heavy metals in muskrats (*Ondatra zibethicus*) with respect to intestinal parasite presence and absence in Pennsylvania, USA, 2018–2019.

Of the nine heavy metals screened for in the subset (n = 120) of livers, six (Cd, Cu, Fe, Mn, Mo, and Zn) were identified above the limit of detection (Table 4; S1 Appendix). The remaining three heavy metals (Pb, Hg and As) were not detected in any of the livers at or above the indicated detection limits. Two muskrats had detectable amounts of Cd in their liver (Table 4). Copper was the only heavy metal to vary in concentration between sexes and females had lower concentrations than males with a mean log_e_-concentration difference of 0.091 (SD = 0.045; Fig 7). Heavy metal concentrations did not vary by water body type (Table 5). The results of our first model indicated that while negative relationships exist between carcass weight and some heavy metal concentration, there was no difference between the slopes or intercepts for each sex. (Fig 8). While there were no differences in the association between heavy metal concentrations and the presence or absence of intestinal parasites, higher mean concentration of Zn, Cu, Mn, and Fe were observed when no parasites were detected (Fig 9). Although no difference was observed in the mean heavy metal concentrations, Mo was the only heavy metal where concentrations were higher in muskrats with active parasite infections (Fig 9). The only chemical detected in muskrat livers during GC/MS and LC/MS testing was phenol (n = 2), however, concentrations were unquantifiable so no connection to disease or morbidity could be inferred.

**Table 4:** Mean (±*SE*) liver concentrations (ppm, wet weight) for heavy metals in both male and female muskrats (*Ondatra zibethicus*) in Pennsylvania, USA, 2018–2019.

Heavy Metal^a^	Total	Male	Female	Range
Pb	ND	ND	ND	ND
	(n = 60)	(n = 60)	(n = 60)	(n = 60)
Mn	3.37±0.27	3.49±*0*.*49*	3.27 (*0*.*27*)	0.64–26
	(n = 112)	(n = 53)	(n = 59)	
Fe	351.61±*18*.*55*	343.02±*24*.*09*	359.32 (*27*.*94*)	120–1500
	(n = 112)	(n = 53)	(n = 59)	
Hg	ND	ND	ND	ND
	(n = 120)	(n = 60)	(n = 60)	
As	ND	ND	ND	ND
	(n = 120)	(n = 60)	(n = 60)	
Mo	0.57±*0*.*01*	0.58±*0*.*02*	0.57 (*0*.*02*)	0.4–0.98
	(n = 81)	(n = 42)	(n = 39)	
Zn	29.43±*0*.*46*	29.45±*0*.*58*	29.41± *0*.*69*	19–51
	(n = 112)	(n = 53)	(n = 59)	
Cu	3.77±*0*.*08*	3.94±*0*.*12*	3.62±*0*.*10*	1.1–7.1
	(n = 112)	(n = 53)	(n = 59)	
Cd	0.007±*0*.*13*	ND	0.43± *0*.*13*	0.00–0.55
	(n = 2)	(n = 60)	(n = 2)	

Means are calculated from samples with detectable amounts of heavy metal concentrations. If no levels of a heavy metal were detected, value is reported as not detected (ND) with respective sample size (n).

^a^ Pb = lead; Mn = manganese; Fe = iron; Hg = mercury; As = arsenic; Mo = molybdenum; Zn = zinc; Cu = copper; Cd = cadmium.

**Table 5:** Mean (±*SE*) liver concentrations (ppm, wet weight) for heavy metals from muskrats (*Ondatra zibethicus*) harvested in different water body types.

Heavy Metal^a^	Creek	Pond	River	Lake	Marsh
	(n = 63)	(n = 40)	(n = 5)	(n = 6)	(n = 5)
Pb	ND	ND	ND	ND	ND
	(n = 63)	(n = 40)	(n = 5)	(n = 6)	(n = 5)
Mn	2.96±0.17	3.98±0.73	4.70±0.77	2.62±0.59	3.14±0.61
	(n = 59)	(n = 37)	(n = 5)	(n = 5)	(n = 5)
Fe	343.90±18.73	386.23±46.45	284.00±19.65	266.00±16.91	364.00±58.02
	(n = 59)	(n = 37)	(n = 5)	(n = 5)	(n = 5)
Hg	ND	ND	ND	ND	ND
	(n = 63)	(n = 40)	(n = 5)	(n = 6)	(n = 5)
As	ND	ND	ND	ND	ND
	(n = 63)	(n = 40)	(n = 5)	(n = 6)	(n = 5)
Mo	0.59±0.02	0.56±0.03	0.40 (0.00)	0.53±0.04	0.61±0.02
	(n = 46)	(n = 25)	(n = 2)	(n = 3)	(n = 4)
Zn	29.98±0.65	28.84±0.73	26.20±1.96	26.80±1.07	33.00±2.75
	(n = 59)	(n = 37)	(n = 5)	(n = 5)	(n = 5)
Cu	3.87±0.07	3.74±0.17	3.02±0.22	3.20±0.32	3.94±0.41
	(n = 59)	(n = 37)	(n = 5)	(n = 5)	(n = 5)
Cd	0.43±0.13	ND	ND	ND	ND
	(n = 2)	(n = 40)	(n = 5)	(n = 6)	(n = 5)

Means are calculated from samples with detectable amounts of heavy metal concentrations. If no levels of a heavy metal were detected, value is reported as not detected (ND) with respective sample size (n).

^a^ Pb = lead; Mn = manganese; Fe = iron; Hg = mercury; As = arsenic; Mo = molybdenum; Zn = zinc; Cu = copper; Cd = cadmium.

## Discussion

Through the volunteer assistance of trappers, we sampled muskrats from throughout all six management regions in Pennsylvania and in several land-cover and water body types (Figs 1 and 2). Males had higher mean carcass weights than females, which aligns with typical sexual dimorphism previously reported in muskrats [30]. Also consistent with previous studies of growth rates, our finding that females with placental scars had higher body weights than those without placental scars provided a crude reference threshold for age class to be classified by body weight, with muskrat with carcass weights under 1000 g being identified as juveniles (Fig 4) [31].

The prevalence and intensity of intestinal helminths was greater in both males and females under 1000 g. These muskrats with lower body weights could represent juveniles, as described above, or animals that were in poor nutritional condition associated with a variety of factors. The lack of pelt and post-mortem condition of the carcasses impacted the ability to accurately evaluate nutritional condition of the muskrats, but none had obvious muscle atrophy suggestive of emaciation (Fig 5). In contrast to our findings, a previous study in Pennsylvania, conducted in 1966, reported higher parasite abundance in older muskrats ($\bar{x}=60.3\;worms$) compared to younger muskrats ($\bar{x}=28.7\;worms$) [32]. Aging techniques are relatively unreliable in this species and should be considered when making comparisons between studies. However, the overall prevalence and abundance we observed in our study were similar to those reported previously in Pennsylvania [32]. With respect to land-cover type, the prevalence of intestinal helminths was moderate in agricultural (41%) and urban (32%) areas compared to forested areas (57%). Our observation of higher parasite prevalence in creeks and ponds matched previous reports in Pennsylvania where creeks had higher prevalence than marshes or rivers [32]. However, our sample size was skewed with most samples coming from creeks and ponds, making comparisons unreliable. The shift observed in parasite abundance between age classes while prevalence in water body types remained similar to previous reports suggests a possible shift in parasite-host interactions. Further investigations beyond our single-season surveillance are warranted to investigate the parasite-host dynamic response to added stressors (e.g., contaminants, climate variability) on juveniles [11].

Based on the passive diagnostic data, Tyzzer’s disease and cysticercosis were two of the most common diagnosed causes of mortality in muskrats in the eastern USA. Both of these diseases have also been reported in existing literature as causes of muskrat morbidity and mortality in North America [5]. We detected *C*. *piliforme* in 11 of the 380 of the trapper-harvested muskrats necropsied, but none of them had gross lesions suggestive of Tyzzer’s disease. Collectively, these results suggest that the bacteria circulate in muskrats in the absence of outbreaks. Tularemia (caused by infection with *F*. *tularensis*) has historically been a cause of large mortality events in muskrats [5]; however, *F*. *tularensis* was not identified as the cause of mortality for any of the 26 muskrat diagnostic cases that were reviewed. Also, *F*. *tularensis* was not identified in any of the trapper-harvested muskrats that were necropsied during active surveillance from 2018 to 2019. These data indicate that muskrats have different roles in the ecologies of tularemia and Tyzzer’s disease. Muskrats appear to be highly susceptible to tularemia and can experience large mortality events, but likely do not harbor *F*. *tularensis* in the absence of disease like they do with *C*. *piliforme*. In the ecology of the disease, muskrats play a role as a transmitter of *F*. *tularensis* to ticks and contamination of water sources that are then used by many other species, including humans [33].

Cysts were found in 46% of the muskrat diagnostic cases and were determined to be the primary cause of morbidity/mortality in five individuals. Four of these cysticercosis cases were caused by *Taenia* spp., which is the genus of tapeworms previously reported from livers of muskrats [5]. In our active surveillance, we detected cysticerci in livers of a similar number (15%) of necropsied muskrats and most (95%) of these were *Hydatigera* spp., with a subset being confirmed as *H*. *taeniaeformis*. This parasite is commonly reported in low prevalence in muskrats and other rodents [5, 34], has been associated with muskrat deaths [35], and is unusual as it can induce hepatic sarcomas [36–38]. The lack of disease in these trapper-harvested muskrats infected with *H*. *taeniaeformis* suggests that disease may not always develop, especially if few parasites are present, they may have been captured during early infections, or other factors may be needed to cause disease.

One of the cysticercosis cases in our passive surveillance was caused by a *Versteria* sp. which was the first detection of this genus in a muskrat [26]. Cysticeri and disease associated with the *Versteria* sp. that we detected in the muskrats has been reported in a captive Bornean orangutan (*Pongo pygmaeus*) in Colorado and a human in Pennsylvania, but neither are natural intermediate hosts [28, 29]. Mink (*Neovison vison*) from Oregon and ermine from Colorado are known definitive hosts [39]. The human report of the *Versteria*. sp. in an adult with common variable immunodeficiency was from western Pennsylvania [28], and although we only detected *Versteria* sp. infections in eastern Pennsylvania, the prevalence was very low so infections in western Pennsylvania could have been missed. Our findings of several muskrat infections confirms they are intermediate hosts. Due to the zoonotic nature of *Versteria* sp., further investigation on the transmission, including potential intermediate and definitive hosts, and impact of this *Versteria* sp. on muskrat health is warranted.

Muskrats have been used as sentinel species for a wide variety of zoonotic pathogens [37], so we tested samples for two parasites (*T*. *gondii*, *Babesia* spp.) that have broader One Health implications. We detected a high prevalence of antibodies to *T*. *gondii* (38%) in the first screen of trapper-harvested muskrats; however, only 1/28 of the secondary PCR screened muskrats was positive for *T*. *gondii*, although infections can be missed by PCR testing of a single tissue and sample. Our prevalence is intermediate to two recent studies on *T*. *gondii* exposure in Illinois (18/30; 60%) and Minnesota (0/70, 0%) [40, 41]. The difference in prevalence could be due to sampling habitats, domestic animal activity at the site, or variable numbers or diversity of intermediate and definitive hosts in the two areas [40]. *T*. *gondii* has been documented as causing disease in sea otters, and there is concern for impacts of *T*. *gondii* on similar species, such as river otters [42]. Muskrats and river otter share similar habitats in Pennsylvania, thus there is a risk of transmission of *T*. *gondii* from feral cats to river otter, as well.

A high percentage of muskrats were positive for a *Sarcocystis* sp. but the actual prevalence is unknown as only muskrats that were seropositive for *T*. *gondii* were tested. Although the species of *Sarcocystis* was not identified in the current study, it was related to other species of rodent intermediate hosts. The significance of this infection for muskrats or other hosts is unknown and a definitive host is unknown. However, considering some species of *Sarcocystis* are recognized pathogens for humans, domestic animals, and wildlife [43], further research is warranted. Interestingly, one muskrat was positive for a *Hammondia* sp. that was distinct from *H*. *hammondi*. Very little is known about this group of coccidians in wildlife, but researchers experimentally established that there was a *Hammondia* sp. that uses mink as definitive hosts and muskrats as intermediate hosts but it is unknown if our sequence is from the same *Hammondia* species [44].

We did not detect Pb, Hg, or As in the subset of 120 muskrat livers analyzed for heavy metals. While the detection limits were 1.0 ppm, this level of sensitivity was believed to be sufficient to detect metal concentrations that might be associated with adverse health effects. While existing reports of mean Pb liver concentrations in muskrats from Ontario, Canada, Washington, USA, and Idaho, USA were within our detection limits (range = 0.27–5.23 ppm), reports for Hg concentrations (range = 0.02–0.22 ppm) were below the detection limit of our toxicant assay [45, 46]. Thus, there is a possibility that Pb, Hg, and As were present in muskrat tissues at low levels, unlikely to be associated with overt disease. Low levels of Cd were detected in two muskrats (Table 4). Cadmium concentrations higher than 0.32 ppm have not been reported in literature on muskrats and no detrimental effects of Cd on muskrat health have been reported [5].

Copper acts as a regulator for Mo, however, when Mo levels become too high and there is not enough Cu to mitigate extreme Mo concentrations, toxic symptoms occur [47]. In a study of an area exposed to heavy metal contamination, Cu concentrations in muskrat livers ranged from 11.51 to 13.77 ppm [45]. In contrast, reports of Cu concentrations in Idaho muskrats were 1.0 to 2.6 ppm [46]. Copper concentrations in our study ranged from 1.1 to 7.1 ppm. In addition, the Mo concentrations we observed (range = 0.4–0.98 ppm) were similar to those in muskrats from Virginia (mean range = 0.73–0.92 ppm) in an area where Mo levels in the environment were also low [48]. This suggests that Mo and Cu levels we observed were normal; however, we do note that Mo was the only heavy metal with higher concentrations in muskrats with intestinal parasites. Previous studies in domestic sheep suggested low dietary Mo supplementation resulted in lower abundance of nematodes and number of infections [49]. Although a similar relationship is observed in trends in Mo concentrations relative to trematode and cestode infections in our muskrats, the potential significance is unclear.

The Zn concentrations we observed (range = 19–51 ppm) fell within ranges previously reported for healthy muskrats (range = 18.4–27.4 ppm) [44]. In comparison, muskrat livers from two contaminated sites in Ontario had mean Zn concentrations of 64.17 and 65.31 ppm. The Mn and Fe concentration levels we observed were also typical of previous studies, except for the Fe concentration of three muskrats exceeding 800 ppm [48]. Although Fe deficiencies are common, Fe toxicosis is not and can result in phosphorus (P) deficiency [50]. We did not screen for P concentrations, and therefore cannot address the possibility of Fe toxicosis in the three muskrats with high Fe concentrations.

There were no differences between water body type and observed heavy metal concentrations, however, sample sizes for rivers, lakes, and marshes were small making statistical testing unreliable (Table 5). While we did detect heavy metal concentrations in muskrat livers, there was no correlation between health, geographical location, or water body type, suggesting that we were unable to detect, if present, the impact of heavy metal contamination on health of muskrat populations. Additionally, GC/MS and LC/MS screens are not comprehensive toxicant screens but can rule out the presence of a number of chemical classes. More specific testing (e.g., testing for many rodenticides or blue green algae toxins) would be beneficial where exposures are suspected based upon history and/or antemortem or postmortem findings.

Overall, we observed low prevalence or negative results for historically common pathogens or parasites of muskrats or other furbearer species, including *T*. *gondii*, *Babesia* spp., and *F*. *tularensis*. *C*. *piliforme* infections, both subclinical and associated with disease, were detected through active and passive surveillance, indicating that this pathogen is circulating in muskrats in Pennsylvania and has the potential to cause mortality. Continued monitoring for *C*. *piliforme* is warranted to detect and anticipate localized outbreaks in the future. Intestinal parasite abundance was comparable to previous studies, however the shift to higher abundance in young muskrats is concerning. Added stressors, such as climate variability, predation, and heavy metal contamination may further increase parasite infection intensity on juvenile muskrats, leading to decreased survival in the future. Future investigations using pelt-primeness to correctly age juveniles are warranted. While our sample was biased to winter collection, our results provide baseline data on exposure of muskrat in Pennsylvania to a suite of pathogens and contaminants. These data can be used to monitor changes in exposure moving forward and can be expanded upon to better understand the impacts of disease on muskrat population declines.

## Supporting information

S1 Appendix Raw data from muskrat necropsies in Pennsylvania from 2018–2019. This table includes pathogen testing results, location of collection, details about collection site, information regarding cysts and helminth detection, as well as the toxicology results.
(XLSX)
